# Evaluation of Visual-Evoked Cerebral Metabolic Rate of Oxygen as a Diagnostic Marker in Multiple Sclerosis

**DOI:** 10.3390/brainsci7060064

**Published:** 2017-06-11

**Authors:** Nicholas A. Hubbard, Yoel Sanchez Araujo, Camila Caballero, Minhui Ouyang, Monroe P. Turner, Lyndahl Himes, Shawheen Faghihahmadabadi, Binu P. Thomas, John Hart, Hao Huang, Darin T. Okuda, Bart Rypma

**Affiliations:** 1 Massachusetts Institute of Technology, Cambridge, MA 02139, USA; ysa@mit.edu (Y.S.A.); csquared@mit.edu (C.C.); 2 Children’s Hospital of Philadelphia, University of Pennsylvania School of Medicine, Philadelphia, PA 19104, USA; ouyangm@email.chop.edu (M.O.); huangh6@email.chop.edu (H.H.); 3 University of Texas at Dallas, Dallas, TX 75080, USA; monroe.p.turner@utdallas.edu (M.P.T.); lyndahl.himes@utdallas.edu (L.H.); shawheen.fahih@utdallas.edu (S.F.); binu.thomas@utsouthwestern.edu (B.P.T.); jhart@utdallas.edu (J.H.); darin.okuda@utsouthwestern.edu (D.T.O.); bart.rypma@utdallas.edu (B.R.); 4 University of Texas Southwestern Medical Center, Advanced Imaging Research Center, Dallas, TX 75235, USA; 5 Department of Neurology and Neurothreapeutics, University of Texas Southwestern Medical Center, Dallas, TX 75235, USA; 6 Department of Psychiatry, University of Texas Southwestern Medical Center, Dallas, TX 75235, USA

**Keywords:** calibrated functional magnetic resonance imaging, multiple sclerosis, diagnosis, visual system, metabolism

## Abstract

A multiple sclerosis (MS) diagnosis often relies upon clinical presentation and qualitative analysis of standard, magnetic resonance brain images. However, the accuracy of MS diagnoses can be improved by utilizing advanced brain imaging methods. We assessed the accuracy of a new neuroimaging marker, visual-evoked cerebral metabolic rate of oxygen (veCMRO_2_), in classifying MS patients and closely age- and sex-matched healthy control (HC) participants. MS patients and HCs underwent calibrated functional magnetic resonance imaging (cfMRI) during a visual stimulation task, diffusion tensor imaging, T_1_- and T_2_-weighted imaging, neuropsychological testing, and completed self-report questionnaires. Using resampling techniques to avoid bias and increase the generalizability of the results, we assessed the accuracy of veCMRO_2_ in classifying MS patients and HCs. veCMRO_2_ classification accuracy was also examined in the context of other evoked visuofunctional measures, white matter microstructural integrity, lesion-based measures from T_2_-weighted imaging, atrophy measures from T_1_-weighted imaging, neuropsychological tests, and self-report assays of clinical symptomology. veCMRO_2_ was significant and within the top 16% of measures (43 total) in classifying MS status using both within-sample (82% accuracy) and out-of-sample (77% accuracy) observations. High accuracy of veCMRO_2_ in classifying MS demonstrated an encouraging first step toward establishing veCMRO_2_ as a neurodiagnostic marker of MS.

## 1. Introduction

Current procedures for diagnosing multiple sclerosis (MS) rely primarily upon clinical presentation and qualitative analysis of standard, medical-grade (e.g., lower resolution) magnetic resonance structural, brain images, e.g., [1]. It has been demonstrated that the diagnostic accuracy of MS can be improved when providers implement advanced neuroimaging techniques and analyses that are not presently common in clinical practice, e.g., [2], see also [3]. Further, research using advanced neuroimaging techniques has demonstrated that these techniques can be more sensitive than their traditional counterparts in detecting subtle changes associated with very early manifestations of MS, e.g., [4,5]. Here, we investigated the accuracy of an advanced neuroimaging technique never before used in MS, calibrated functional magnetic resonance imaging (cfMRI), to classify MS patients and closely age- and sex-matched healthy controls (HCs). Specifically, we focused our analyses upon the ability of a new neuroimaging marker, visual-evoked cerebral metabolic rate of oxygen (veCMRO_2_), to accurately discriminate between MS patients and HCs. 

cfMRI is a relatively new neuroimaging technique that capitalizes upon established relationships between blood-oxygen-level dependent (BOLD) signal and cerebral blood flow (CBF) in order to estimate steady-state, oxygen metabolism [6,7] see [8]. The technique gets its name from the use of a BOLD-calibration parameter, often acquired during a gas-inhalation challenge. The CMRO_2_ metric permitted by cfMRI offers several advantages over the more commonly used BOLD signal. First, CMRO_2_ offers physiological specificity. CMRO_2_ represents a true physiological process, oxygen metabolism, whereas BOLD reflects a confluence of processes and as such, is physiologically non-specific. Second, calibration-derived CMRO_2_ is strongly tied to electrical and chemical neural activity, e.g., [9,10,11,12,13,14,15], whereas an appreciable component of BOLD signal is unexplained by neural activity, e.g., [16,17,18,19,20], see [21], but see [9]. Finally, CMRO_2_ measures are not dependent upon the hemodynamic assumptions of BOLD, making them optimal measures of brain function in populations with atypical hemodynamics, like MS, e.g., [22,23], see [24].

Evaluating CMRO_2_ as a diagnostic marker of MS is particularly relevant for these patients because MS is associated with changes to neurometabolism. Neuroimaging research has produced considerable evidence of altered neurometabolism in MS, e.g., [25,26,27,28,29]. In one study, Ge and colleagues [30] demonstrated decreases in brain-wide resting CMRO_2_ for MS patients relative to HCs. Some neuroimaging studies have shown that neurometabolic alterations were related to white matter macrostructural (i.e., lesions, e.g., [30]) or microstructural damage in MS, e.g., [27,28]. For example, magnetic resonance spectroscopy in centrum semiovale white matter has shown that *N*-acetylaspertate (NAA) and NAA: creatine ratios were strongly related to diffusion-weighted indices of white matter structural integrity in MS patients [27].

It is intuitive that MS patients would show differences in in vivo neurometabolism when considering that postmortem analyses have revealed extensive alterations to the mitochondria in lesioned and non-lesioned MS neural tissue [31,32,33], see [34,35,36]. For instance, Singhal and colleagues [33] found decreases in postmortem NAA, a partial marker of neuronal respiratory capacity, and decreases in electron transport subunit proteins across lesioned and non-lesioned MS grey matter, relative to matched control participants’ grey matter. Taken together, the results of postmortem and in vivo neuroimaging studies demonstrate that neurometabolic alterations are generally featured in MS.

Evaluating veCMRO_2_ should also be particularly relevant as a diagnostic marker of MS because MS is marked by alterations to the neural substrate of the visual system, see [37,38,39,40] see also [5]. The use of advanced imaging techniques such as high-resolution structural brain imaging, optical coherence tomography (OCT), functional magnetic resonance imaging (fMRI), and diffusion tensor imaging (DTI) has revealed that visual system alterations exist even in MS patients without visual disturbances or a history of optic neuritis (a clinical syndrome closely linked to MS and marked by visual impairment and visual pathway insult). Indeed, there are MS-related structural alterations to both early (e.g., retinae) and later (e.g., optic radiations) portions of the afferent visual pathway, and alterations to visuocortical activity in patients without a history of optic neuritis see [39]. For instance, Alshowaier and colleagues [41] used electroencephalogram recordings to show that MS patients without a history of optic neuritis demonstrated delayed inion channel, multifocal visual-evoked electrical potentials relative to age- and sex-matched HCs. Previous work in our laboratory has also revealed alterations to visual cortex BOLD signal during visual stimulation in MS patients with normal or corrected-to-normal vision compared to matched HCs [42], see also [43]. Together, structural and functional imaging results suggest that changes to the visual system are a robust marker of MS pathology. 

MS is associated with changes to neurometabolism and alterations to the neural substrate of the visual system. Thus, visual-evoked oxygen metabolism signals in visual cortex (i.e., veCMRO_2_) should be a diagnostically relevant marker of MS. We assessed the extent to which veCMRO_2_ signals could be used to discriminate between MS patients and HCs. The classification accuracy of veCMRO_2_ was examined in the context of other variables commonly assayed in MS, including measures of neurological insult (e.g., gross lesion volume, parenchymal atrophy), neuropsychological change (e.g., Brief Repeatable Battery of Neuropsychological Tests [44]), and self-report symptom measures (e.g., subjective fatigue). We tested the extent to which veCMRO_2_, and these other measures, could classify MS status using both within-sample and out-of-sample observations. 

## 2. Materials and Methods

### 2.1. Participants

Participants between the ages of 18 and 65 were recruited for this study. Participants were required to be free of MR-contraindicators, concurrent substance abuse, have normal or corrected-to-normal vision, and speak fluent English. Because study procedures included a gas-inhalation challenge (see Section 2.4), participant selection was limited to non-smokers. Participants did not have histories of respiratory or pulmonary problems, cerebral vascular issues, or cardiac problems. Participants were required to have a score greater than 21 on the telephone interview for cognitive status [45]. Thirty-one participants in total met the inclusion criteria. 

Twelve MS patients meeting the above criteria were recruited from the Clinical Center for Multiple Sclerosis at the University of Texas Southwestern Medical Center. Eleven patients had a diagnosis of relapsing-remitting MS and one patient had a diagnosis of secondary-progressive MS. Patients were required to be at least 1 month past their most recent exacerbation and their last corticosteroid treatment. Patients were recruited who did not report a history of optic neuritis. Patients without a history of optic neuritis were specifically selected so as to limit additional variability from attributed to severe, anterior visual pathway damage/dysfunction (e.g., such as that resulting from conduction block) and potential visual impairment. All MS patients’ vision was normal or corrected-to-normal. Two patients withdrew or declined to undergo the gas challenge (total *n* = 10). 

Nineteen HC participants were recruited from the Dallas-Fort Worth Metroplex via email, posted flyers, and word-of-mouth. These participants were evaluated for the general inclusion/exclusion criteria described above. Three HCs did not undergo the scanning protocol because of exclusions discovered after study enrollment (e.g., concussion history revealed after pre-screening, incidental MR finding). Two HCs withdrew or declined to undergo the gas challenge. During imaging processing (see Section 2.5), one HC’s functional images failed to appropriately register to their anatomical image after multiple attempts, so this person was excluded. Thirteen HCs (*n* = 13) remained for subsequent analyses. These participants were closely age- and sex-matched to the MS patients (see Table 1).

### 2.2. Study Procedures

Study procedures were approved by the University of Texas Southwestern Medical Center Institutional Review Board. Recruitment numbers were approximated based upon previous research showing sufficient power to demonstrate group changes in calibrated fMRI (cfMRI) contrasts with similar sample sizes [22,23]. Participants meeting inclusion criteria were asked to refrain from caffeine use at least two hours before their scheduled appointment time, e.g., [47]. They were also asked not to consume alcohol on the same calendar day before their scheduled appointment. Participants gave written informed consent before undergoing procedures and were compensated for their time. Participants underwent functional and structural neuroimaging on a Philips 3-Tesla magnet (Philips Medical Systems, Best, The Netherlands) with an 8-channel SENSE radiofrequency head coil. Foam padding was placed around the head to minimize motion during MRI scan acquisition. Participants completed standard neuropsychological tests (e.g., Brief Repeatable Battery of Neuropsychological tests [44]) and self-report measures regarding their general health and symptomology (i.e., SF-36 [48], Modified Fatigue Impact Scale (MFIS, [49]); see Table 2 for a complete list of model variables).

### 2.3. cfMRI Parameters and Theory

Dual-echo pseudocontinuous arterial spin labeling (pCASL) and BOLD images (together referred to as dual-echo images) were acquired using an interleaved echo scanning protocol see [7,52]. Together, the perfusion (Echo 1) and BOLD-weighted (Echo 2) images along with biophysical modeling procedures allowed for estimation of CMRO_2_ and a neural-vascular coupling coefficient (*n*, see [8]) associated with steady-state, neural stimulation [5,7]. One task run of dual-echo imaging data and one gas-challenge run of dual-echo imaging data were collected using the following parameters: Echo 1: labeling duration 1650 ms, labeling flip angle 18°, labeling gap = 63.5 mm, 3.44 × 3.44 × 5 mm voxel, repetition time (TR) = 4000 ms, echo time (TE) = 14 ms, 1525 ms post-label delay, 0 mm slice gap. Echo 2: 90° flip angle, 3.44 × 3.44 × 5 mm voxel, TR = 4000 ms, TE = 40 ms, 0 mm slice gap. Total scan time for the visual stimulation task = 600 s (72 dual-echo dynamics). Total scan time for the gas challenge = 624 s (75 dual-echo dynamics).

Estimations of CMRO*2* and *n* were based upon the Davis model of BOLD signal change [6,7]:

(1) $$\frac{\Delta S}{S_{0}}=M\left({\left(1-\frac{\Delta \mathrm{CBF}}{{\mathrm{CBF}}_{0}}\right)}^{\propto -\beta }{\left(\frac{\Delta {\mathrm{CMRO}}_{2}}{{\mathrm{CMRO}}_{2|0}}\right)}^{\beta }\right)$$

where ∆x/x_0_ denotes a change from baseline, α is an empirically derived constant linking cerebral blood flow and cerebral blood volume, and β is an empirically derived constant related to vascular exchange and susceptibility of deoxyhemoglobin at specific field strengths (e.g., [53,54,55]). We assumed α = 0.38 [56] and β = 1.3 [52]; these values were chosen because they have been shown to be sensitive to group differences in neurophysiology [22,23]. Also, these values have previously demonstrated group-equivalence in the estimation of *M*, e.g., [22,23]. *M* is a subject-specific scaling factor dependent upon the washout resting deoxyhemoglobin see [8]. *M* was estimated in each participant, using the gas challenge detailed below.

The measurement of BOLD, CBF, and *M* allows for the estimation of CMRO_2_. Here, ∆CMRO_2_ reflects the visual task-related change in neurometabolism of oxygen from resting baseline:

(2) $$\frac{{\Delta \mathrm{CMRO}}_{2}}{\;{\mathrm{CMRO}}_{2|0}}=\;{\left(1-\frac{\frac{\Delta \mathrm{BOLD}}{{\mathrm{BOLD}}_{0}}}{M}\right)}^{1/\beta }{\left(\frac{\Delta \mathrm{CBF}}{{\mathrm{CBF}}_{0}}\right)}^{1-\;\alpha /\beta }$$

where ∆x/x_0_ reflects percent change of signal during task compared to resting baseline. With the estimation of ∆CMRO_2_, *n*, may also be estimated:

(3) $$n=\;\frac{\frac{\Delta \mathrm{CBF}}{{\mathrm{CBF}}_{0}}}{\frac{\Delta {\mathrm{CMRO}}_{2}}{{\mathrm{CMRO}}_{2|0}}}$$

thus, *n* reflects per unit output of ∆CBF per unit input of ∆CMRO_2_ see [8]. 

### 2.4. cfMRI Task and Gas Challenge

Participants completed a visual stimulation task during dual-echo task imaging. This task was chosen for two reasons. First, differences in the functional response to visual stimulation have been observed in MS visual cortex see [42,57]. Second, because this task required minimal effort, group differences in performance were not expected to be a factor. 

**Figure 1:**
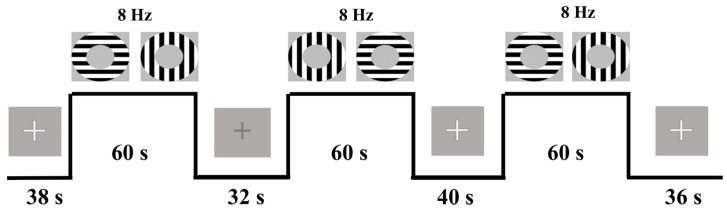
Example of three-trial visual stimulation task. Participants viewed a fixation cross at the center of the screen. This cross changed color at jittered intervals throughout task. Rest periods were also jittered. Continuous stimulation blocks lasted 60 s with 0° to 90° flickering annuli (at 8 Hz). *Note:* fixation cross was presented during task and rest periods however it cannot be seen in the task example periods here.

Participants were trained on the task before entering the MR environment. During the task, participants focused on a fixation cross at the center of their visual field. Participants were required to respond via bilateral, thumb-button press when a change in the luminance of the fixation cross occurred. This task was used in order to control the center of the participants’ visual field [22,23,58]. Change in luminance was jittered and occurred every 2, 3, 4, or 6 s. Visual stimulation occurred in a block format. There were 6 visual stimulation task blocks consisting of 60 s of continual annulus flickering in the participants’ near-foveal visual field. Annuli alternated at orthogonal orientations (0 to 90°) to avoid neural adaptation [58]. Alterations occurred at a constant frequency of 8 Hz because both electrochemical neural activity and BOLD signal have been shown to peak at this frequency, potentially yielding the greatest signal-to-noise estimates, e.g., [59,60]. Rest blocks were jittered at 32, 34, 36, 38, and 40 s intervals (see Figure 1).

Participants also completed a gas-challenge in order to estimate *M*. Participants breathed 4 min of room air (~0.03% CO_2_: 21% O_2_: 78% N_2_) and 6 min of an iso-oxic, CO_2_ solution (5% CO_2_: 21% O_2_: 74% N_2_) during dual-echo imaging. Each participant was fitted with a two-way, non-rebreathing valve/mouthpiece and a nose clip. Baseline end-tidal CO_2_ (EtCO_2_), O_2_ saturation, breath rate, and heart rate measures were collected. After the 4 min of room air breathing, a valve was opened to release the CO_2_ solution from a Douglas airbag which then flowed into the participants’ breathing apparatus [22,23]. The CO_2_ inhalation lasted 6 min. 

Hypercapnic challenge, via the inhaled 5% CO_2_ solution, increases global CBF, but probably has no or a minimal depressant effect on oxygen metabolism, e.g., [61,62,63]. Hypercapnia acts to wash out local baseline concentrations of deoxyhemoglobin, yielding a local maximum estimate of resting BOLD signal. Potential changes to oxygen metabolism due hypercapnic challenge have not been shown to appreciably alter the estimation of *M* as relationships between hypercapnia-derived *M* and *M* derived from non-hypercapnic techniques show high correspondence [64].

### 2.5. cfMRI Processing

Task and gas-challenge Echo 1 and Echo 2 data were processed in analysis of functional neuroimages (AFNI [65]) and the Functional MRI of the Brain Software Library (FSL [66]). Data were transformed into cardinal planes. Anomalous data points in each voxel time series were then attenuated using an interpolation method based upon the average signal. Data were volume registered to correct for motion to the fourth functional volume of each dataset’s (task or gas challenge) Echo 2 sequence using a heptic polynomial interpolation method. CBF was estimated from Echo 1 images using the surround subtraction method [67]. Dual-echo BOLD data were also interpolated by pairwise averaging of temporally adjacent images.

For the visual stimulation task, Echo 2 data were linearly registered (12 degrees-of-freedom) to each participant’s anatomical data using AFNI’s *align_epi_anay.py* program. The transformation matrix from this registration was then applied to Echo 1 data, placing these two datasets in the same space. For gas-challenge data, a binary mask was created for functional voxels in Echo 2 to aid in co-registration. This mask was then registered to the respective participant’s anatomical space using the *align_epi_anay.py* program. Gas-challenge Echo 2 and Echo 1 data were also aligned to the mask which was registered in native anatomical space. After alignment, Echoes 1 and 2 data from both the visual task and gas challenge were visually inspected for registration errors. One HC participant failed to register correctly after multiple attempts and was discarded from further analyses. Echoes 1 and 2 data from the visual task and gas challenge were then spatially smoothed using a Gaussian kernel (FWHM = 8 mm) and high-pass filtered (0.0039 Hz).

Preprocessed data from Echoes 1 and 2 in the visual stimulation task were analyzed via generalized linear modeling of task versus rest periods using a boxcar reference function. This modeling quantified task-related CBF and BOLD changes from baseline. BOLD and CBF beta-values were scaled to each voxel’s resting baseline signal and were multiplied by 100, yielding percent signal change estimates from baseline (∆BOLD and ∆CBF). Data were averaged from a visual (functional) region of interest (ROI) comprised of overlapping ∆BOLD and ∆CBF suprathreshold signals within occipital lobe (see Structural and Functional ROI; [22,23]). ∆BOLD, ∆CBF, ∆CMRO_2_, and n results extracted from the functional region of interest were taken as the visual-evoked signals (i.e., veBOLD, veCBF, veCMRO_2_, and ve*n*). 

**Figure 2:**
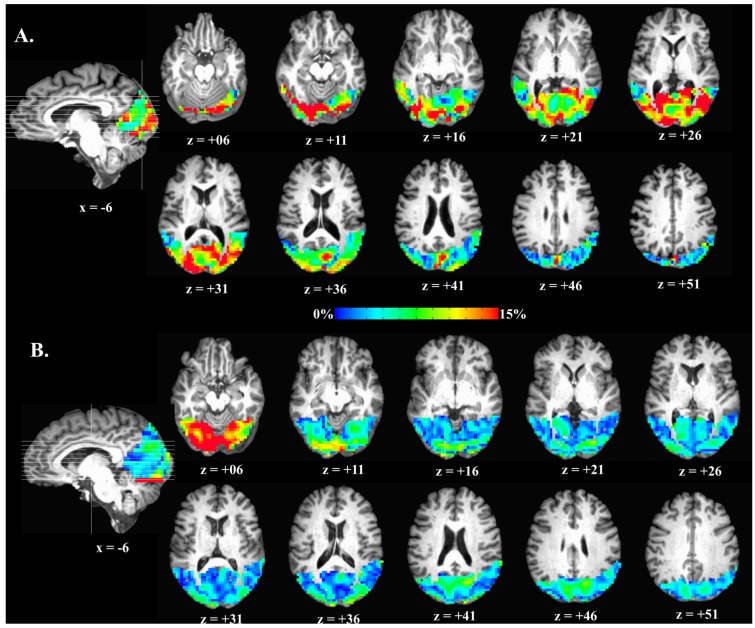
Examples of oxygen metabolism changes (∆CMRO_2_)in occipital lobe. (**A**) HC ∆CMRO_2_; (**B**) MS patient ∆CMRO_2_. x = right-left, z = superior-inferior.

For the gas challenge, resting baseline BOLD and CBF signals during room air breathing were averaged for each voxel time-series (BOLD_0_ and CBF_0_). The first two minutes of hypercapnia BOLD and CBF time-series were discarded to allow participants’ blood flow to stabilize on the CO_2_ solution, e.g., [22,23]. The last four minutes of hypercapnia BOLD and CBF time-series were averaged to yield BOLD_hc_ and CBF_hc_ respectively. Average values were extracted from a functional region of interest (see Structural and Functional ROI) using overlapping BOLD_hc_ and CBF_hc_ suprathreshold signals within occipital lobe, and were used to calculate *M*, using the following equation:

(4) $$M=\frac{\frac{{\mathrm{BOLD}}_{\mathrm{hc}}-\;{\mathrm{BOLD}}_{0}}{{\mathrm{BOLD}}_{0}}}{\left(1-{\left(1+\frac{{\mathrm{CBF}}_{\mathrm{hc}}-\;{\mathrm{CBF}}_{0}}{{\mathrm{CBF}}_{0}}\right)}^{\alpha -\beta }\right)}$$

where (x_hc_−x_0_)/x_0_ reflects percent change in signal from normocapnic to hypercapnic states, normalized by the signals during normocapnia and multiplied by 100. Once *M* was estimated, ∆CMRO_2_ and *n* were also estimated (see Equations (2) and (3); see Figure 2) within a functional region of interest (see Structural and Functional ROI).

### 2.6. Structural and Functional ROIs

First, the magnetization-prepared rapid acquisition gradient-echo (MPRAGE) data were processed to create a native-space, occipital ROI. The skull was removed using an automated command, separating parenchyma and cerebral spinal fluid from the skull. An intensity based automated segmentation algorithm was used to delineate primarily white matter, grey matter, and cerebral spinal fluid voxels yielding a partial volume estimate of each tissue type, for each voxel. A grey matter mask was then created, retaining voxels with only a greater than or equal to grey matter partial volume estimate of 80%. A structural ROI of occipital lobe was manually delineated on each participant’s MPRAGE image. These were drawn in native space because native space analyses tend to allow for more sensitive patient-control contrasts [68]. The structural ROI was drawn using gyral and sulcal landmarks and encompassed most of occipital cortex including calcarine sulcus, cuneus, and occipital portions of lingual gyrus. Several anatomical landmarks were used in the demarcation of this ROI (parieto-occipital sulcus, occipital pole, pre-occipital notch). Within the anatomically defined occipital lobe, only voxels with partial volume estimates of grey matter (≥80%) were retained. These final masks were down-sampled to the functional voxel size.

**Figure 3:**
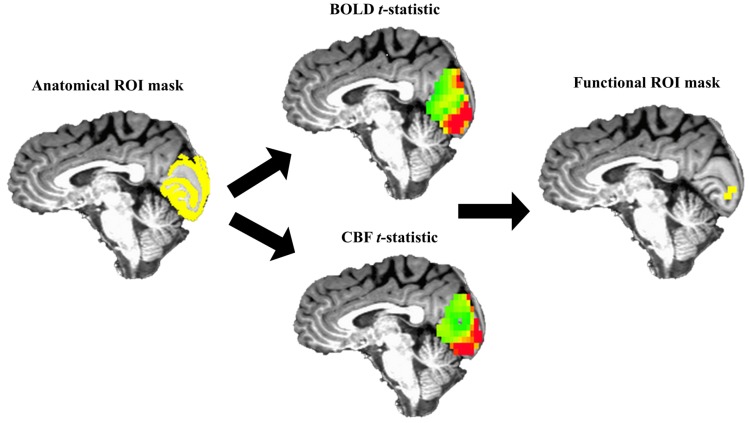
Graphical overview of masking procedure. For each participant, their top 5%, overlapping BOLD and CBF *t*-statistics (middle) within the anatomical ROI (left, yellow) were used to create the functional ROI mask (right, yellow). Functional measures (veBOLD, veCBF, veCMRO_2_, and ve*n*) were extracted from each participant’s functional ROI mask.

A visual task functional ROI was created within the structural ROI described above to estimate veBOLD, veCBF, veCMRO_2_, and ve*n* (see Figure 3). This procedure eschewed noise from inactive voxels, e.g., [68]. Voxels comprising each participant’s functional ROI were the overlapping top 5% of BOLD and top 5% of CBF *t*-values obtained from the generalized model, within the structural ROI. This ensured that average veBOLD and veCBF estimates were being derived from the same, task-responsive voxels and that veCMRO_2_ and ve*n* were derived in voxels with both CBF and BOLD task-related increases (see Figure 3). 

veCMRO_2_ was calculated voxel-wise within the functional ROI using ∆BOLD, ∆CBF, *M* (which was extracted from functional ROI described below). ve*n* was then calculated similarly. The final product of these analyses was average positive veBOLD, veCBF, and veCMRO_2_, and ve*n* extracted from the functional ROI (see Figure 3).

Because the gas challenge data differed in occipital coverage compared to the visual task data, *M* was estimated ex situ. To create a functional ROI for the gas challenge, ∆BOLD_hc_/BOLD_0_ and ∆CBF_hc_/CBF_0_ maps were thresholded and extracted from the structural ROI detailed above. The criteria for retention of a voxel within these maps required that the voxel was within the top 15% (top 20% for one participant) of ∆BOLD_hc_/BOLD_0_ and ∆CBF_hc_/CBF_0_ voxels in the structural ROI, and that these ∆BOLD_hc_/BOLD_0_ and ∆CBF_hc_/CBF_0_ voxels overlapped. This procedure ensured complementary maximum ∆BOLD_hc_/BOLD_0_ and ∆CBF_hc_/CBF_0_ signals in the retained voxels. Average ∆BOLD_hc_/BOLD_0_ and ∆CBF_hc_/CBF_0_ signals were extracted from this ROI and *M* was calculated (see Equation (4)).

### 2.7. Structural Images

**Figure 4:**
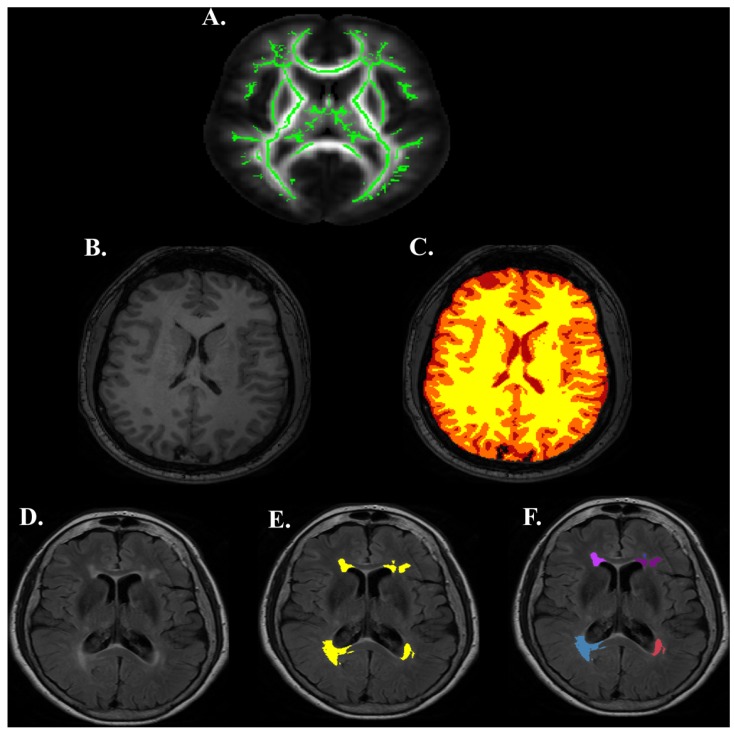
Diffusion and Structural Image Processing Examples. (**A**) Diffusion tensor imaging white matter skeleton. (**B**) T_1_ image. (**C**) T_1_ image segmented into white matter (yellow), grey matter (orange), and cerebral spinal fluid (red) using SIENAX. (**D**) T_2_-FLAIR image. (**E**) Lesions demarcated (yellow) on T_2_-FLAIR image used for calculating lesion burden. (**F**) Spatially distinct lesions demarcated on T_2_-FLAIR image.

One T_1_-weighted MPRAGE image was acquired for each participant: 160 slices, TE = 3.7 ms, repetition time TR = 8.1 ms, sagittal slice orientation, 1 × 1 × 1 mm^3^ voxel, 12° flip angle. SIENAX [15,69] was used to obtain measures of grey matter, white matter, and total brain volume normalized by participant’s head size. This technique uses partial volume estimation to calculate volume of differing tissue types (see Figure 4B,C). Further, this technique takes into account lesioned tissue, as demarcated by lesion masks (see below), in order to avoid misclassification of this tissue. The final products of these analyses were scaled estimates of each participant’s grey matter, white matter, and total brain volume (mm^3^).

A T_2_ fluid attenuated inversion recovery (FLAIR) scan was also acquired for each participant: 33 slices, TE = 125 ms, TR = 11,000 ms, no slice gap, transverse slice orientation, 0.45 × 0.45 × 5.00 mm^3^ voxel, 120° refocusing angle. FLAIR images were used to estimate the extent of gross lesion burden for each participant. Hyperintense voxels were demarcated using in-house MATLAB code based upon slice-wise, signal intensity (i.e., voxels that were ≥1.25 SD over the slice mean intensity). Next, lesions were manually delineated from the hyperintense tissue by two trained researchers (L.H., S.F.). Manual delineation ruled out false positives in lesion classification due to fat signals, motion, ventricular edge effects, skull, or signal inhomogeneites [70]. Lesion burden was estimated by extracting the number of voxels that were demarcated by the automated and manual procedures. Inter-rater agreement of lesion burden was calculated using a Dice ratio (κ) of the lesion burden estimates made by the two researchers on a sample of several subjects [71]. After the researchers were trained on lesion classification, inter-rater agreement was found to be high, κ = 0.89; where κ > 0.70 is generally thought to reflect excellent inter-rater agreement [72]. Lesion burden was quantified using absolute (total mm^3^ of lesioned tissue; see Figure 4E) and relative scales (percent of total mm^3^ of lesioned tissue scaled by uncorrected white matter volume in mm^3^). Spatially distinct lesion count was also obtained by counting the number of non-touching lesions for each subject (see Figure 4F), e.g., [73]. A lesion was required to have at least 3 mm^3^ volume in order to be added to the total lesion count. Thus, the final products of these analyses were absolute lesion volume, relative lesion volume, and spatially distinct lesion count. 

### 2.8. Diffusion Images

DTI images were acquired using a single-shot, echo-planar imaging sequence with a Sensitivity Encoding parallel imaging scheme (reduction factor = 2.3), 112 × 112 matrix, field of view = 224 × 224 mm^2^ (nominal resolution of 2 mm), 65 slices (0 mm gap), slice thickness = 2 mm, TR = 7.78 s, TE = 97 ms. The diffusion weighting was encoded along 30 independent orientations [74] and the b value was 1000 s/mm^2^. Imaging time was 5 min and 15 s. Two HCs did not undergo DTI (*n*HC = 11).

Automatic Image Registration [75] was performed on raw diffusion-weighted images to correct distortion caused by eddy currents. Six elements of the 3 × 3 diffusion tensor were determined by multivariate least-squares fitting. The tensor was diagonalized to obtain three eigenvalues (λ_1–3_) and eigenvectors (v_1–3_). Standard tensor fitting was conducted with DTIStudio [76] to generate the most common DTI-derived diffusion characteristics, fractional anisotropy (FA), axial diffusivity (AD), mean diffusivity (MD), and radial diffusivity (RD).

DTI measurements were obtained at the skeletons of the white matter using FSL [77] to alleviate partial volume effects with tract-based spatial statistics (see Figure 4F–H) [77]. Participant FA maps were registered nonlinearly to the EVE single-subject FA template [78,79,80] for better alignment with a digital white matter atlas (JHU ICBM-DTI-81) [81]. Registered FA maps of all subjects were averaged to generate a mean FA map, from which an FA skeleton mask was created. Skeletonized FA images of all subjects were obtained by projecting the registered FA images onto the mean FA skeleton mask. Skeletonized AD, MD, and RD metrics were obtained by applying the same registration, projection, and skeletonization procedures. We extracted skeleton-wide averages of each DTI metric (i.e., AD, FA, MD, RD), wherein an average of each metric is calculated across all voxels within the white matter skeleton (see Figure 4A). 

### 2.9. Statistical Analyses

All analyses were performed on distributions free of outliers (≥±2 SD from group mean for simple group comparisons, ≥±3 MAD from group median for classification modeling see [82]). Binary logistic regression was used for classifying MS status. A description of model variables can be found in Table 2. The accuracies of these models were computed as the proportion of correct classification outcomes over all outcomes. Accuracy was chosen as the metric of interest because it combines sensitivity and specificity in binary classification analysis by taking into account both true positives and true negatives relative to all outcomes. We used resampling-based hypothesis testing to examine both within-sample and out-of-sample classification of patient status see [83]. Because we used relatively conservative analytic techniques, inherently reducing the likelihood of Type I error and increasing the generalizability of our results, the criterion for a rejection of the null hypothesis was not corrected for multiple comparisons and all models were evaluated at the field-standard α = 0.05. We also denote which hypothesis tests survived Benjamini-Hotchberg correction (Table 4; Figure 7). 

Within-sample classification analyses obtained bias-corrected and accelerated (BCa) bootstrapped-resampled (B = 10,000) 95% confidence intervals of the accuracy of binary logistic regression models. The BCa procedure was used because it is robust to both skewness and sampling bias in the bootstrap distribution [84]. To avoid unstable classification, we stratified all resamples to match the original sample’s constitution of patients and controls, 56.5% and 43.5%, respectively. If the BCa-derived 95% confidence interval did not contain a value at or below 0.50 (binary chance), this would demonstrate the measure’s accuracy was significantly greater than chance to classify MS patients and HCs.

Out-of-sample classification analyses used a leave-one-out cross-validation approach [85]. This technique used training and sample iterations to test the ability of the model derived from the training set to predict an observation in the test (out-of-sample) set, thus, circumventing problems of sample bias, model over fitting, and lending a true predictive element to these analyses. Briefly, the leave-one-out cross validation (LOOCV) approach fitted N models, where N was proportional to our sample size. Each model was trained on N-1 samples and then the accuracy of the training model was assessed on the left-out sample. The N accuracies were then averaged to attain a representative and generalizable measure of the average out-of-sample classification accuracy. Permutation based *p*-values (5000 permutations) were computed to assess the significance of the LOOCV-derived accuracy statistics. The test permuted patient status labels and recomputed the accuracy of the model at each iteration, thus building the null distribution. The *p*-values were calculated from the percentage of the accuracy estimates of the permuted samples that were better than actual LOOCV-derived accuracy statistic of each model. This procedure was slightly modified according to Ojala and Garriga [86].

## 3. Results

### 3.1. Visual Task Performance

MS patients (92.75 ± 1.11%) did not significantly differ from HCs (94.86 ± 0.44%) on accuracy on the visual stimulation secondary task, *t*(10.54) = −1.76, *p* = 0.108. Patients (492.06 ms ± 31.15) also did not significantly differ from HCs (487.19 ms ± 24.10) on their average correct response time to press the button on the secondary task, *t*(16.22) = 0.12, *p* = 0.903. 

### 3.2. Group Physiology, Cerebrovascular Response to Gas Challenge, and M

MS and HCs did not significantly differ in breath rate, end-tidal CO_2_, heart rate, or O_2_ saturation at baseline or during CO_2_ solution breathing (all ps > 0.05; see Table 3). We tested whether MS patients differed in their CBF response to the CO_2_ solution ((CBF_hc_−CBF_0_)/CBF_0_) and *M* in their respective gas challenge ROIs within occipital lobe see [87]. MS patients did not significantly differ in CBF response to the CO_2_ solution (167.48 ± 19.8%) compared to HCs (146.90 ± 14.64%), *t*(15.70) = 0.83, *p* = 0.417. MS patients (3.88 ± 0.48%) did not significantly differ in M compared to HCs (5.11 ± 0.39%), *t*(18.90) = −1.98, *p* = 0.062.

### 3.3. Group Comparisons on Visual Task cfMRI Measures

**Figure 5:**
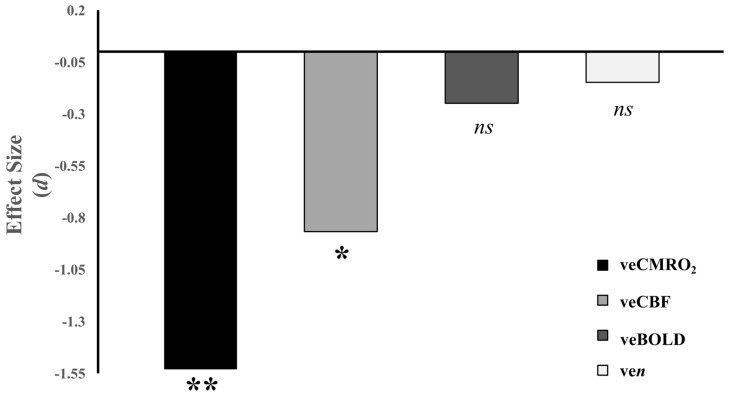
Effect sizes of group contrasts on calibrated functional magnetic imaging measures. Effect sizes reflect Cohen’s *d*. *ns* = non-significant effect, *p* > 0.05; * *p* < 0.05; ** *p* < 0.01.

MS patients (1.12 ± 0.77%) did not significantly differ from HCs (1.18 ± 0.66%) on veBOLD response to visual stimulation, *t*(19.18) = −0.60, *p* = 0.555. MS patients (4.08 ± 0.35) did not show significant changes in ve*n* compared to HCs (4.23 ± 0.23), *t*(16.16) = −0.35, *p* = 0.731. MS patients (48.06 ± 12.58%) had significant decreases in veCBF compared to HCs (92.68 ± 17.29%), *t*(19.76) = −2.09, *p* = 0.050. MS patients (9.59 ± 0.90%) also showed significant decreases in veCMRO_2_ compared to HCs (17.85 ± 1.97%), *t*(16.45) = −3.81, *p* = 0.002 (see Figure 5).

### 3.4. Within-Sample Classification Analyses

**Figure 6:**
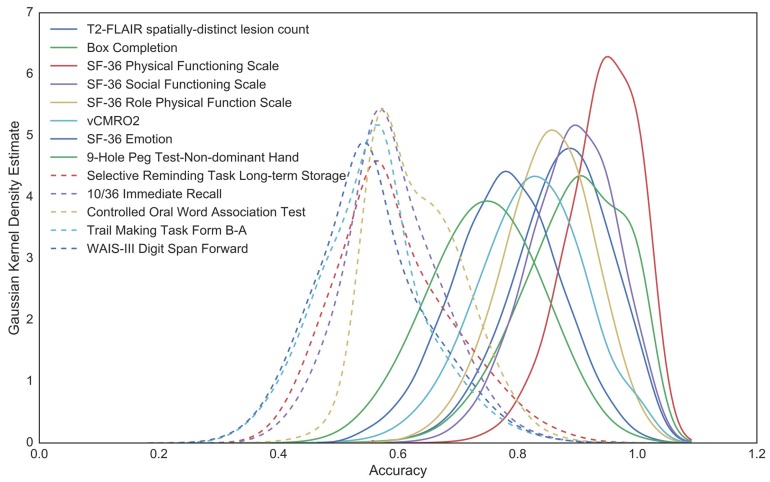
Smoothed density estimates of BCa-bootstap distributions. Distributions of significant (solid lines) and bottom 5 (dashed lines) within-sample predictors of MS status are illustrated. *Note*: because of smoothing, tails of distributions may exceed 1.

Measures are ranked on original accuracy and presented in Table 4. Accuracy and smoothed density distributions for the significant and bottom 5 measures can be found in Figure 6. 

### 3.5. Out-of-Sample Classification Analyses

**Figure 7:**
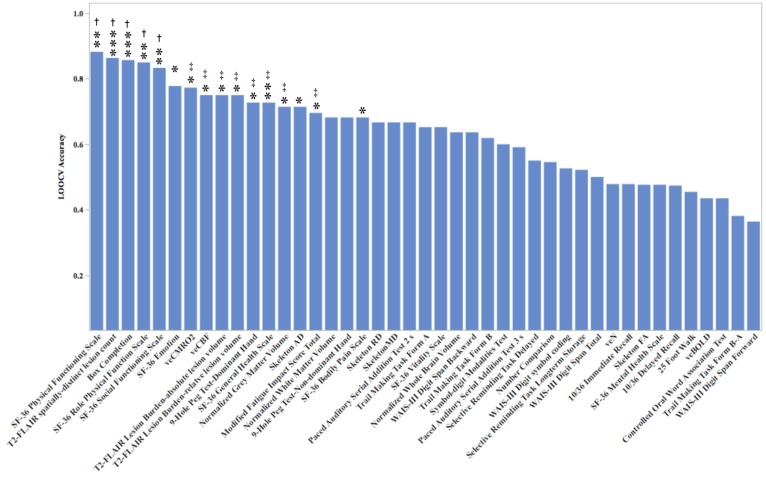
Leave-one-out cross-validation (LOOCV) out-of-sample classification accuracy of each model. * *p* < 0.05; ** = *p* < 0.01; *** = *p* < 0.001. † *p*-value also significant using Benjamini-Hotchberg correction (*p* < 0.05). ‡ *p*-value marginally significant using Benjamini-Hotchberg correction (*p* < 0.10).

Predictors presented in Figure 7 are ranked on LOOCV-derived accuracy. 

## 4. Discussion

In the present study, we used a neuroimaging approach novel to MS research (cfMRI) to assess the accuracy of veCMRO_2_ in classifying MS patients and closely age- and sex-matched HC participants. MS patients showed similar responses to HCs in veBOLD and ve*n*, however showed decreased veCBF and a pronounced decrease in veCMRO_2_ relative to HCs. Groups were similar on visual task performance and on physiological measures pertaining to the CO_2_ challenge, indicating that potential MS-related changes in physiological response to carbon dioxide, e.g., [87] or visual attention were not likely contributors to group CMRO_2_ differences. Within-sample classification analyses demonstrated that veCMRO_2_ was significant and one of the top measures to accurately classify MS status, discriminating between MS patients and HCs with exceptional accuracy (82%). Results also showed that within-sample classification accuracy by veCMRO_2_ was comparable to neuroimaging measures often used to gauge MS pathology, such as T_2_-FLAIR lesion burden (80% accuracy) and T_1_ grey matter volume (81% accuracy). veCMRO_2_ was also significantly accurate in MS classification using out-of-sample observations (77% accuracy). The use of such out-of-sample modeling afforded a predictive element to this study and demonstrated that veCMRO_2_ can accurately classify new observations of MS and HC participants, offering support for its potential diagnostic utility. 

One question that arises from these results is whether veCMRO_2_ can add predictive value over other advanced imaging techniques not studied here. For instance, measurements of multifocal visual-evoked potentials have been of great interest to the MS research community. This technique, which uses visual stimulation and electroencephalogram signals in occipital channels proximal to the inion has been demonstrated to (1) more sensitively and specifically detect visual abnormalities in MS eyes relative to other visual-system measurements [88], (2) predict conversion to an MS diagnosis in persons with optic neuritis [89], and (3) relate to the extent of MS-related damage to visual white matter tracts [41]. Not surprisingly, this technique can also accurately discriminate between MS patients and HCs, e.g., [90]. For example, one study showed that measurements gathered from multifocal visual-evoked potentials were on average 74.76% accurate (range: 62.7%–96.1%) in classifying within-sample observations of MS patients without optic neuritis and HCs ([90], average calculated from Figure 5 and Figure 6, pp. 910–911). We can compare these figures with the within-sample accuracy of veCMRO_2_ observed here (82%). This suggests that veCMRO_2_ accuracy is in about the same range as multifocal evoked potentials. However, it performs appreciably better than the average multifocal evoked potential measure. Future research directly comparing veCMRO_2_ to electroencephalogram and other measures is necessary to more faithfully adjudicate claims about the relative performance of this technique.

A second avenue for future research could involve examining whether the integration of evoked CMRO_2_ from other neural systems could maximize MS classification accuracy. Here, we showed significant decreases in MS patients’ veCMRO_2_ relative to HCs. This variable was also largely accurate in the prediction of MS status. We looked at veCMRO_2_ specifically because of robust alterations to the visual system in MS see [37,38,39,40]. However, because (1) mitochondrial alterations are found in multiple forms of neural tissue in MS [31,33] and (2) global brain decreases in oxygen metabolism have been found in MS patients relative to HCs [30], it is likely that evoked CMRO_2_ is affected in other neural systems as well. Our work and others’ have shown altered patterns of brain activity in MS patients in motor, e.g., [42,91,92] and association cortices [43,93,94,95], see [96]. It is possible that the addition of measures of evoked CMRO_2_ in these areas could lend improvements in the accuracy of MS classification. One advantage of the cfMRI approach over other advanced imaging approaches in MS, like OCT or visual-evoked potentials, is that this technique can specifically and simultaneously assay multiple neural systems. Work underway in our laboratories is examining the extent to which evoked motor and executive system CMRO_2_ differs between MS patients and age- and sex-matched healthy HCs, and whether these changes, along with veCMRO_2_, can help build optimal neurodiagnostic models of MS.

The utility of imaging biomarkers in MS is not limited to assisting in diagnosis see [97]. For instance, OCT measures have been shown to be effective in predicting brain atrophy and visual acuity loss in MS see [38]. The retinal nerve fiber thickness and macular volume measures from OCT might also be useful in differentiating different subtypes of MS [98]. Other imaging-based measures, such as T_2_-lesion burden, have shown prognostic ability by prediction of future MS disability, e.g., [99], see also [100,101,102]. One potential avenue for future research is to evaluate the use of oxygen metabolism signals in MS prognosis. For example, Ge and colleagues’ [27] research showed that lower resting brain-wide levels of oxygen metabolism were associated with both increased neurological disability and increased lesion burden in MS patients. Although these findings were cross-sectional, they suggested that oxygen metabolism could be a marker of the trajectory of disease course. To wit, future longitudinal work should examine whether measures of oxygen metabolism in early MS can predict future disease progression cf. [89]. veCMRO_2_ or resting oxygen metabolic markers could also be evaluated for their abilities to predict the transition from risk states (such as clinically or radiologically isolated syndrome) to clinically definite MS see [100,102,103].

A recent wave of findings related to metabolic dysfunction in MS has led to metabolic hypotheses to explain the pathophysiology of MS see [34,35,36]. For instance, Paling and colleagues furthered an energy failure hypothesis of the pathophysiology of MS [35,104]. These authors postulated a link between white matter damage and energy demand in MS, wherein this damage causes neuroenergetic demand to exceed the supply of metabolic substrate. This hypothesis is largely consistent with the findings of the present study, wherein the observed relative decrease in veCBF (the supply of oxygen and glucose) in MS might have limited the neurometabolic response (veCMRO_2_) relative to HCs. Further, issues of oxygen extraction due to mitochondrial damage/dysfunction could have also contributed to the relative decrease in veCMRO_2_ for MS patients relative to HCs see [34,35,36].

Imaging techniques here and elsewhere have produced convincing biomarkers of MS see [38,97,100]. However, MS is a complex, multifaceted disease. Thus, it is not surprising that our results revealed a diverse array of measures that were accurate in classifying MS patients and HCs. The goal of this work was to examine the ability of a new marker (veCMRO_2_) to accurately classify MS. However, a truly prodigious advance in MS diagnostics will likely evolve from models that combine many relevant factors. It is possible that a “gold-standard” model of MS diagnostics would contain information about evoked CMRO_2_, along with other information like lesion count, self-reported symptomology, neuropsychological performance, and potentially other strong associates of MS not examined here (e.g., low-contrast letter acuity performance see [105], oligoclonal band status [106], retinal nerve fiber layer thickness see [38]). For instance, research from the Alzheimer’s Disease Neuroimaging Initiative showed that a complement of multimodal neuroimaging, cerebrospinal fluid proteins, along with standard clinical evaluations allow for optimal prediction of conversion from mild cognitive impairment to Alzheimer’s disease [107]; see also [108] for application in psychiatry. 

## 5. Conclusions

This study was the first to apply cfMRI in an MS sample. Presently, the intricacies of cfMRI acquisition and post-acquisition processing probably hinder it from having an immediate impact upon routine diagnosis or tracking of MS. However, acquisition continues to be optimized and research is showing promise toward eliminating the gas-challenge component of this method, see [8], which should increase the ease of cfMRI administration and the diversity of patients in which it can be applied. With contemporary research highlighting the importance of neurometabolism in the pathophysiology of MS and continued optimization of this technique, cfMRI shows promise as a translational diagnostic/prognostic tool for MS. 

Our findings demonstrated that veCMRO_2_ was accurate in classifying both within- and out-of-sample observations of MS patients and HCs. Out-of-sample analyses suggested that predictive models using veCMRO_2_ could be useful in MS diagnostics and potentially new cases of MS. Although out-of-sample analyses provide confidence in the generalizability of our findings, larger, independent samples are desirable to confirm the robustness of these effects. However, the present findings represent an encouraging first step in realizing the diagnostic relevance of veCMRO_2_ in MS.

## Figures and Tables

**Table 1:** Group Characteristics.

	MS	HC	*p*
Age	50.10 (3.35)	50.77 (3.35)	0.885 ^a^
MFIS	39.10 (7.62)	20.54 (4.57)	0.046 ^a^
Sex (% female)	90.00%	84.62%	0.704 ^b^
TICS Score	27.00 (0.82)	28.08 (1.43)	0.520 ^a^
Age of MS Onset	38.67 (2.42)	-	-
Disease Duration	118.80 (19.32)	-	-
Last Flare-up	28.60 (11.32)	-	-
Neurological Disability Score	15.70 (3.71)	-	-
Disease Modifying Therapies			
Dalfampridine	50%	-	-
Dimethyl fumarate	10%	-	-
Fingolimod	20%	-	-
Glatiramer acetate	10%	-	-

Mean (SEM). Age in years. MFIS = modified fatigue impact score total. Sex in percent female. TICS score = telephone interview for cognitive status score. Age of MS onset in years. Disease duration and last flare-up in months. Neurological disability score measured by self-report [46]. Disease modifying therapies represent percent of participants reporting use of therapy. ^a^ *p*-value based upon independent samples *t*-test. ^b^ *p*-value based upon Pearson *χ*^2^.

**Table 2:** Predictor Variables.

Predictor (Units if Available)	Predictor Category	What Predictor Measures
Normalized Grey Matter Volume (mm^3^)	MR Image	Total grey matter volume normalized to skull
Normalized White Matter Volume (mm^3^)	MR Image	Total white matter volume normalized to skull
Normalized Whole Brain Volume (mm^3^)	MR Image	Total brain volume normalized to skull
Skeleton AD (mm^2^/s)	MR Image	Diffusion along primary diffusion axis
Skeleton FA (proportion)	MR Image	Proportion of anisotropic diffusion
Skeleton MD (mm^2^/s)	MR Image	Average Diffusion in primary diffusion axes
Skeleton RD (mm^2^/s)	MR Image	Diffusion orthogonal to primary diffusion axis
T_2_-FLAIR Lesion Burden-absolute lesion volume (mm^3^)	MR Image	Total volume of lesioned brain tissue
T_2_-FLAIR Lesion Burden-relative lesion volume (%)	MR Image	Total lesioned brain tissue relative to total white matter volume
T_2_-FLAIR spatially distinct lesion count	MR Image	Total number of spatially distinct lesions
veBOLD (% signal change)	MR Image	Visual cortex BOLD response to visual stimulation task
veCBF (% signal change)	MR Image	Visual cortex CBF response to visual stimulation task
veCMRO_2_ (% signal change)	MR Image	Visual cortex CMRO_2_ response to visual stimulation task
ve*n* (proportion)	MR Image	Visual cortex neural-vascular coupling
10/36 Delayed Recall (total correct after 15 min)	Neuropsych	Visuospatial memory/learning and delayed recall
10/36 Immediate Recall (total correct)	Neuropsych	Visuospatial memory/learning
25 Foot Walk (s)	Neuropsych	Walking ability and gait speed
9-Hole Peg Test-Dominant Hand (s)	Neuropsych	Finger and hand dexterity
9-Hole Peg Test-Non-dominant Hand (s)	Neuropsych	Finger and hand dexterity
Box Completion (items completed)	Neuropsych	Motor control
Controlled Oral Word Association Test (total correct)	Neuropsych	Verbal association fluency
Number Comparison (items completed)	Neuropsych	Processing speed
Paced Auditory Serial Addition Test 2 (% correct)	Neuropsych	Processing speed and selective/sustained attention
Paced Auditory Serial Addition Test 3 (% correct)	Neuropsych	Processing speed and selective/sustained attention
Selective Reminding Task Delayed (items recalled)	Neuropsych	Verbal learning and memory
Selective Reminding Task Long-term Storage (items recalled)	Neuropsych	Verbal learning and long-term memory
Symbol-digit Modalities Test (items completed)	Neuropsych	Sustained attention and concentration
Trail Making Task Form A (s)	Neuropsych	Visual search, attention, mental flexibility, and motor function
Trail Making Task Form B (s)	Neuropsych	Visual search, attention, mental flexibility, and motor function
Trail Making Task Form B-A (s)	Neuropsych	Visual search, attention, mental flexibility, and motor function
WAIS-III Digit Span Backward (items completed)	Neuropsych	Short-term, working memory
WAIS-III Digit Span Forward (items completed)	Neuropsych	Short-term, working memory
WAIS-III Digit Span Total (items completed)	Neuropsych	Short-term, working memory
WAIS-III Digit symbol coding (items completed)	Neuropsych	Performance subtest of WAIS
Modified Fatigue Impact Score	Symptoms	Fatigue symptomology
SF-36 Bodily Pain Scale	Symptoms	General measure of bodily pain
SF-36 Emotion	Symptoms	Role limitations due to emotional problems
SF-36 General Health Scale	Symptoms	General measure of health wellbeing
SF-36 Mental Health Scale	Symptoms	General measure of mental health
SF-36 Physical Functioning Scale	Symptoms	General measure of physical functioning
SF-36 Role Physical Function Scale	Symptoms	Role limitations due to physical problems
SF-36 Social Functioning Scale	Symptoms	General measure of social functioning
SF-36 Vitality Scale	Symptoms	General measure of energy/fatigue

FLAIR = Fluid-attenuated inversion recovery. WAIS = Wechsler adult intelligent scale. SF-36 = Short-form health survey. MR Image = magnetic resonance image; Neuropsych = neuropsychological test; Symptoms = self-report general health and symptom measures. Explanations of neuropsychological tests and symptom measures taken from [44,48,50,51].

**Table 3:** Sample Physiological Data.

	MS	HC	*p*
Baseline			
Breath Rate	11.20 (1.00)	10.25 (0.79)	0.747 ^a^
EtCO_2_	42.70 (1.81)	39.23 (0.74)	0.101 ^b^
Heart Rate	66.90 (2.38)	72.08 (3.18)	0.207 ^b^
SpO_2_	98.10% (0.35%)	97.85% (0.32%)	0.596 ^b^
5% CO_2_			
Breath Rate	13.35 (1.28)	15.42 (1.07)	0.236 ^c^
EtCO_2_	48.95 (1.45)	49.06 (0.64)	0.950 ^c^
Heart Rate	69.67 (2.38)	75.04 (2.60)	0.147 ^d^
SpO_2_	97.58% (0.39%)	98.20% (0.20%)	0.139 ^d^

Mean (SEM). Breath Rate in breaths per minute. EtCO_2_ = end-tidal CO_2_ in mmHg. Heart Rate in beats per minute. SpO_2_ = peripheral oxygen saturation in percent hemoglobin saturation. *p*-values were based on independent samples. ^a^ 22 degrees-of-freedom; ^b^ 21 degrees-of-freedom; ^c^ 16 degrees-of-freedom; ^d^ 17 degrees-of-freedom.

**Table 4:** Accuracy and 95% Confidence Limits of Within-Sample Classification Analyses.

Predictor	Predictor Accuracy	95% LCL	95% UCL	Significant
SF-36 Physical Functioning Scale	0.94	0.65	1.00	Yes †
SF-36 Social Functioning Scale	0.89	0.61	0.94	Yes †
T_2_-FLAIR spatially distinct lesion count	0.86	0.57	0.95	Yes †
Box Completion	0.86	0.52	0.95	Yes †
SF-36 Role Physical Function Scale	0.85	0.60	0.95	Yes †
veCMRO_2_	0.82	0.55	0.91	Yes ‡
Normalized Grey Matter Volume	0.81	0.43	0.95	No ‡
T_2_-FLAIR Lesion Burden-absolute lesion volume	0.80	0.50	0.90	No ‡
T_2_-FLAIR Lesion Burden-relative lesion volume	0.80	0.50	0.90	No ‡
SF-36 Emotion	0.78	0.56	0.89	Yes
9-Hole Peg Test-Non-dominant Hand	0.77	0.55	0.91	Yes ‡
SF-36 General Health Scale	0.77	0.50	0.86	No ‡
veCBF	0.75	0.45	0.85	No ‡
Normalized Whole Brain Volume	0.73	0.45	0.86	No
9-Hole Peg Test-Dominant Hand	0.73	0.50	0.82	No
SF-36 Bodily Pain Scale	0.73	0.45	0.86	No
Skeleton AD	0.71	0.43	0.81	No
Skeleton MD	0.71	0.48	0.86	No
Paced Auditory Serial Addition Test 2 s	0.71	0.48	0.86	No
Modified Fatigue Impact Score Total	0.70	0.43	0.78	No ‡
Normalized White Matter Volume	0.68	0.45	0.82	No
Paced Auditory Serial Addition Test 3 s	0.68	0.45	0.82	No
Skeleton RD	0.67	0.48	0.76	No
Trail Making Task Form A	0.65	0.43	0.78	No
SF-36 Vitality Scale	0.65	0.43	0.74	No
25 Foot Walk	0.64	0.50	0.77	No
WAIS-III Digit Span Backward	0.64	0.41	0.77	No
WAIS-III Digit Span Total	0.64	0.41	0.82	No
10/36 Delayed Recall	0.63	0.42	0.74	No
Trail Making Task Form B	0.62	0.33	0.76	No
SF-36 Mental Health Scale	0.62	0.38	0.62	No
veBOLD	0.61	0.48	0.78	No
Selective Reminding Task Delayed	0.60	0.35	0.60	No
Symbol-digit Modalities Test	0.60	0.30	0.70	No
Number Comparison	0.59	0.36	0.68	No
WAIS-III Digit symbol coding	0.58	0.37	0.58	No
Skeleton FA	0.57	0.37	0.67	No
ve*n*	0.57	0.39	0.52	No
Selective Reminding Task Long-term Storage	0.57	0.35	0.70	No
Controlled Oral Word Association Test	0.57	0.35	0.57	No
10/36 Immediate Recall	0.52	0.30	0.57	No
WAIS-III Digit Span Forward	0.50	0.27	0.55	No
Trail Making Task Form B-A	0.48	0.29	0.52	No

LCL = lower confidence limit. UCL = upper confidence limit. Confidence limits based upon 10,000 iteration BCa-corrected bootstrapping procedure.Yes = 95% confidence interval (CI) does not contain 0.50; No = 95% CI contains 0.50. Note: that the original parameter estimates do not necessarily need to lie within the 95% CI of the BCa-corrected, empirically derived distributions. † permutation *p*-value significant using Benjamini-Hotchberg correction (*p* < 0.05). ‡ permutation *p*-value marginally significant using Benjamini-Hotchberg correction (*p* < 0.10).
